# Selectivity of Entomopathogenic Fungi to *Chrysoperla externa* (Neuroptera: Chrysopidae)

**DOI:** 10.3390/insects11100716

**Published:** 2020-10-19

**Authors:** Pamella Mingotti Dias, Elisângela de Souza Loureiro, Luis Gustavo Amorim Pessoa, Gabriel Luiz Reis Devoz, Gilson Bárbaro Barbosa Junior, Allan Macali Werner, Acacio Aparecido Navarrete, Paulo Eduardo Teodoro

**Affiliations:** 1 Graduate Program in Entomology and Biodiversity Conservation, Federal University of Grande Dourados, Dourados 79804-970, Brazil; pamellamingotti@hotmail.com; 2 Agronomy, Federal University of Mato Grosso do Sul, Chapadão do Sul 79560-000, Brazil; luis.pessoa@ufms.br (L.G.A.P.); gabriel.devoz@gmail.com (G.L.R.D.); paulo.teodoro@ufms.br (P.E.T.); 3 Graduate Program in Agronomy, Area of Concentration in Crop Science, Federal University of Mato Grosso do Sul, Chapadão do Sul 79560-000, Brazil; gilson.barbarobarbosa@gmail.com (G.B.B.J.); allanwerner@hotmail.com (A.M.W.); acacionavarrete@gmail.com (A.A.N.)

**Keywords:** Hypocreales, entomopathogens, microbial control, entomophagous, predator, green lacewing, integrated pest management, sustainability

## Abstract

**Simple Summary:**

Agricultural crop systems have adopted integrated management as a model of success in pest control; however, chemical control is still prioritized. The use of pesticides incorrectly and excessively has provided a reduction in natural enemies, selection of resistant populations and resurgence of pests. *Chrysoperla externa* is a predator found in several regions in Brazil that preys on different pest insects. Entomopathogenic fungi *Beauveria* and *Metarhizium* also stand out for causing epizootics on pests. Both predators and entomopathogens can simultaneously act as pest control; thus, verifying the selectivity of entomopathogenic fungi to predators increases the potential for biological control through synergism and conservation of natural enemies in the agroecosystem. Considering the control potential of these different biological control agents, in this study we evaluated the selectivity of *Beauveria bassiana*, *Metarhizium anisopliae* and *Metarhizium rileyi* to the larvae of this predator. The results provide evidence that the biological development of larvae of *C. externa* is not influenced by the entomopathogenic fungus. These species of fungi can be recommended, aiming at a management of populations of arthropod pests, with low effect on *C. externa* when it is present in the agrosystem.

**Abstract:**

We aimed to evaluate the selectivity of entomopathogenic fungi to larvae of *Chrysoperla externa* (Neuroptera: Chrysopidae). For this purpose, *Beauveria bassiana* (strain ESALQ PL63), *Metarhizium anisopliae* (strain ESALQ E9) and *Metarhizium rileyi* (strain UFMS 03) were assessed at different concentrations (1 × 10^7^, 1 × 10^8^ and 1 × 10^9^ conidia mL^−1^). The control treatment consisted of distilled water and Tween80 0.01. The treatments were applied with a Potter spray tower using two different methodologies: direct application (DA) and dry film (DF). Up to 96 h after application, no treatment provided a larval mortality above 3%. After 120 h, only *B. bassiana* induced significant mortality in all instars, with rates of 26%, 17% and 10% for first, second and third instar larval periods, respectively. There was no difference regarding to the application method or concentration of conidia. The percentage of individuals that revealed changes in the length of the larval and pupal periods varied among different treatments with entomopathogenic fungi and control treatments, application methodologies and concentrations. Despite *B. bassiana* revealing a higher mortality than *M. anisopliae* and *M. rileyi* on larvae of *C. externa*, these three entomopathogenic fungi may be used in association with *C. externa* for sustainable pest management.

## 1. Introduction

Neotropical fauna of green lacewings present high diversity, with 82 genera [1]. The *Chrysoperla externa* species (Hagen, 1861) (Neuroptera: Chrysopidae) is commonly found in Brazil, with reports of occurrence in different regions [2]. Studies have shown the effectiveness of this predatory species in the biological control of pest arthropods (such as aphids and whiteflies), eggs and small caterpillars, as well as in field and controlled conditions for cotton, wheat, sorghum, citrus, peach and melon crops [3,4,5,6,7,8,9,10,11,12,13,14]. In addition to prey generalism, *C. externa* has aroused interest in biological control research due to the ease of its mass production in the laboratory and its environmental adaptability [10,11].

Entomopathogenic fungi are important regulators of pest arthropods due to the natural action of these microorganisms through natural epizootic diseases [15]. In the system of integrated pest management (IPM), the fungi belonging to the genera *Beauveria*, *Metarhizium* and *Isaria* are widely used in the biological control of several arthropod pests [16,17]. The adoption of IPM in agricultural production systems has been considered a success [18,19]; however, chemical control is still a priority due to the desire to lower production costs and improve the lack of suitable information about the association of management tools applicable in the fields [19,20].

The incorrect and excessive use of pesticides has led to reductions of natural enemies, selections of resistant populations and resurgences of pests [20,21,22]. Selective phytosanitary products may intervene in the development and survival of entomophagous insects and entomopathogenic microorganisms [23,24,25]. There are reports of the negative effects of pesticides at all stages of development of *C. externa* [26,27,28,29,30]. The selectivity of the entomopathogenic fungi concerning green lacewings raises the potential for biological control by the synergism and conservation of natural enemies in the agroecosystem. The entomopathogenic fungi used to control pests in Brazil has been shown to be selective in the species of green lacewings *C. externa* and *Ceraeochrysa cincta* (Neuroptera: Chrysopidae) [29,30,31,32,33].

Due to the information presented on the Chrysopidae, we would like to obtain better insight into the actions of the entomopathogenic fungi *Beauveria bassiana* (strain ESALQ PL63), *Metarhizium anisopliae* (strain ESALQ E9) and *Metarhizium rileyi* (strain UFMS 03) based on the mortality and duration of the first-, second- and third-instar (larval) periods of *C. externa* at 24, 48, 72, 96 and 120 h after fungi application.

## 2. Materials and Methods

### 2.1. Obtaining Fungi

The fungi *B. bassiana* (strain ESALQ PL63) and *M. anisopliae* (strain ESALQ E9) were obtained from the microbiological insecticides BOVERIL and METARRIL, respectively. However, because no commercial formulas contain *M. rileyi* (strain UFMS 03), this isolate was produced following the methods described by Dias et al. [34]. Sabouraud media, temperature and relative humidity (25 ± 1 °C, 70 ± 10% of relative humidity), with a photoperiod of 12 h (light/dark = L:D), were also used to obtain the isolate of *M. rileyi*. To prepare fungal suspensions, the isolates were diluted using sterile distilled water that contained 0.01% (*v*/*v*) Tween 80, and the conidia were counted using a Neubauer chamber to standardize the concentrations.

### 2.2. Obtaining and Rearing Chrysoperla externa

Adults of *C. externa* were collected in corn crops at the Federal University of Mato Grosso do Sul, Campus of Chapadão do Sul, MS, Brazil, where chemicals had not been sprayed. After this, adults were kept in 10-cm-diameter PVC (polyvinyl chloride) cages (height 23 cm), which were lined with white bond paper for oviposition, with the lower end supported by styrofoam lined with paper and the upper end sealed with VOILE fabric. Insects were fed an artificial diet of brewer’s yeast and honey, as described by Ribeiro [35]. The diet was provided using an adapted feeder made up of a soft sponge containing a paste diet over the top base and a bottom part inserted into a 10-mL cylindrical tube with distilled water, according to the methodology described by Dias et al. [36].

Eggs were removed from the oviposition substrate using either scissors or a fine plastic comb at 24 h after oviposition. The eggs (10 each) were then transferred to 500-mL plastic containers that had been properly sanitized using 70% alcohol and 12 h of UV irradiation. The containers (i.e., cages) contained strips of paper to create a refuge for the larvae against cannibalism. Larvae were fed with sterilized eggs of *Anagasta kuehniella* (Zeller) (Lepidoptera: Pyralidae) daily until sufficient numbers of larvae were available for bioassays [10]. All insects were stored in an acclimatized room (temperature of 25 ± 1 °C, 70 ± 10% relative humidity (RH), and photoperiod of 12 h (L:D)).

### 2.3. Effects of the Entomopathogenic Fungi in the Chrysoperla externa Larvae

Under controlled conditions with a temperature of 25 ± 1 °C, 70 ± 10% RH, and a photoperiod of 12 h (L:D), larvae of first, second and third instars of *C. externa* were separated and placed in Petri dishes (Supplementary Figure S1A–C). With a Potter spray tower adjusted to the pressure of 1.5 MPa, 2 mL of the following treatments were given: a control comprising sterilized distilled water and 0.01% (*v*/*v*) Tween 80 (Sigma-Aldrich, Saint Louis, USA); and *B. bassiana* (strain ESALQ PL63), *M. anisopliae* (strain ESALQ E9) and *M. rileyi* (strain UFMS 03), each at a concentrations of 1 × 10^7^, 1 × 10^8^ and 1 × 10^9^ conidia mL^−1^. Into all fungal suspensions, 0.01% (*v*/*v*) of Tween 80 was added (Supplementary Figure S1D). Two methodologies were used: the first was the direct application (DA) of the treatments on the larvae of first, second and third instars inside Petri dishes (Anidrol, Diadema, Brazil) (called arenas) [37] (Supplementary Figure S1E); the second methodology involved dry film (DF), in which the treatments were applied on the arenas, with the insertion of larvae after drying the excess moisture (Supplementary Figure S1F), according to the model proposed by the International Organization for Biological and Integrated Control of Harmful Animals and Plants, Western Palearctic Regional Section (IOBC/WPRS) [38,39]. After the treatments were applied, all insects were fed sterilized eggs of *A. kuehniella* (Zeller) (Lepidoptera: Pyralidae) daily.

After the application and drying, the Petri dishes were sealed with plastic film and treated insects were placed within the biological oxygen demand (BOD) (Eletrolab, Campinas, Brazil) chamber under the following conditions: temperature 25 ± 1 °C, relative humidity 70 ± 10% and a photoperiod of 12 h (L:D). Larvae were supplied with a standard diet (*A. kuehniella* eggs) according to the nutritional needs in each instar [40].

The larval mortality and percentage of insects at each growing stage were determined by 24, 48, 72, 96 and 120 growing hours after application (HAA). For confirmation of larval mortality, the dead bodies were superficially disinfected with 70% alcohol (Copersucar SA, São Paulo, Brazil) and sterile distilled water, three consecutive times, then placed in a humid chamber [31] under the following conditions: temperature 25 ± 1 °C, relative humidity 70 ± 10% and a photoperiod of 12 h (L:D), (Supplementary Figure S2A–C).

For the third instar (larval) period that survived treatments, the evaluations were extended to 168 HAA, counting the percentage of individuals who had reached the pre-pupal and pupal stages. The duration (days) of these stages was also evaluated. The pupal viability was evaluated by the emergence of adults.

Adult viability was evaluated until 24 h after their emergence. Insects were considered viable when they had regular morphological specifications: whole antennae, compound eyes, full abdomen, total opening of the wings and a length between 20 and 23 mm. This visual scale was adopted based on information available in the literature [11] (Supplementary Figure S3A–C).

### 2.4. Statistical Analysis

The experimental design was completely randomized, with four replications in a factorial scheme. The first factor comprised four fungal strains (P = products); the second factor was represented by four concentrations (C = control treatment, 1 × 10^7^, 1 × 10^8^ and 1 × 10^9^ conidia mL^−1^); and the third factor involved two application methods (A = direct application (DA) and dry film (DF)).

A Shapiro–Wilk test was used to verify the normality of the residuals for all evaluated variables. After verifying the normal distribution adjustment at 5% probability, we performed an analysis of variance (ANOVA). In the three-way ANOVA, the isolated effects of P, C and A were tested, as well as their interactions (P × A, P × C, A × C and P × A × C). When we detected significance, we applied a Tukey test at 5% probability for the comparison of means. All analyses were performed with the free software Rbio (Federal University of Viçosa, Viçosa, Brazil) [41].

## 3. Results

### 3.1. Mortality of Chrysoperla externa Larvae

In all instars, larvae of *C. externa* showed significantly higher susceptibility (*p*-value < 0.05) to the (P) at 120 HAA. Survival of first instar (larval) period from 24 to 120 HAA was not influenced by the application methods (A), concentrations (C) or interactions (P × A, P × C, A × C and P × A × C) (*p*-value > 0.05; Supplementary Tables S1–S3).

**Figure 1:**
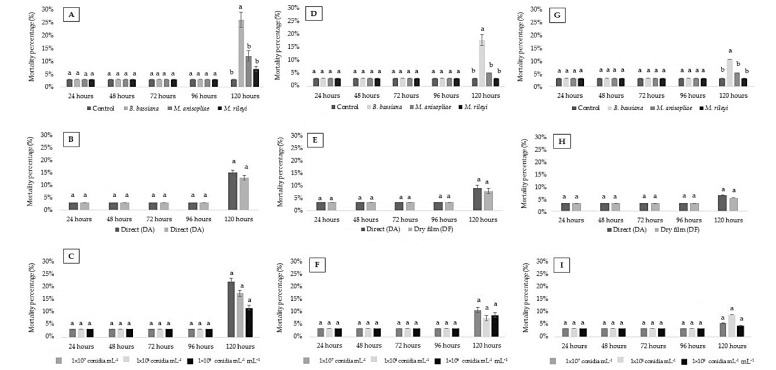
Mortality percentage (% ± standard error) of the first, second and third *Chrysoperla externa* larvae after each period of application of entomopathogenic fungi *Beauveria bassiana*, *Metarhizium anisopliae* and *Metarhizium rileyi* (T: 25 ± 1 °C, RH 70 ± 10% and 12 h photoperiod (light/dark)). (**A**–**C**) First instance mortality factor = P (products), A (application methods) and C (concentrations), respectively. (**D**–**F**) Second instar mortality factor = P (products), A (application methods) and C (concentrations), respectively. (**G**–**I**) Third instar mortality factor = P (products), A (application METHODS) and C (concentrations), respectively. Values (%) followed by equal letters at each assessment time do not differ from each other according to a Tukey test at 5% probability.

From 24 to 96 HAA, the percentage of dead insects (3%) observed for the first instar (larval) period of *C. externa* was not influenced by the isolate factors (P, A and C) or its interactions (P × A, P × C, A × C and P × A × C). Only *B. bassiana* showed slightly higher mortality (26%) than other strains at 120 HAA (Figure 1A).

For factor A, mortality was recorded as 15% and 13% at 120 HAA for the first instar (larval) period exposed to the methodologies DA and DF, respectively. Mortality observed at 120 HAA was 19%, 15% and 10% for concentrations in the order of 1 × 10^7^, 1 × 10^8^ and 1 × 10^9^ conidia mL^−1^, respectively (Figure 1B,C).

The mortality by *B. bassiana* (P) was higher for the second instar (17%) at 120 HHA; for factor A, mortalities of 8% for DA and 7% for DF were observed, while for factor C mortalities of 10%, 7% and 8% were observed relative to concentrations 1 × 10^7^, 1 × 10^8^ and 1 × 10^9^ conidia mL^−1^, respectively (Figure 1D–F).

For the third instar, the highest mortality shown by *B. bassiana* at 120 HAA was 10%. Factors A and C recorded mortalities of 6% in DA and 5% in DF; and factor C recorded mortalities of 5%, 8% and 4% in concentrations of 1 × 10^7^, 1 × 10^8^ and 1 × 10^9^ conidia mL^−1^, respectively (Figure 1G–I).

Confirmed mortality was observed in all dead bodies of larvae infected with *B. bassiana* (Supplementary Figure S4A–C).

### 3.2. Change of Larval State of C. externa Larvae

For the first instar of *C. externa,* there was a significant effect of the treatments applied (P), with significant differences from 48 to 120 HAA. There were differences among the concentrations (C) only in the evaluation of 120 HAA (*p*-value < 0.05; Supplementary Table S4).

For the second instar, variations in change of the larval stage were observed at 72 and 96 HAA, with significant differences only for the products (P) (*p* < 0.05; Supplementary Table S5).

For the third instar of *C. externa,* there was a significant effect of the applied treatments (P), application methods (A) and concentrations (C) at 72 HAA, but only for the applied treatments (P) and concentrations (C) at 120 HAA (*p*-value < 0.05; Supplementary Table S6).

**Figure 2:**
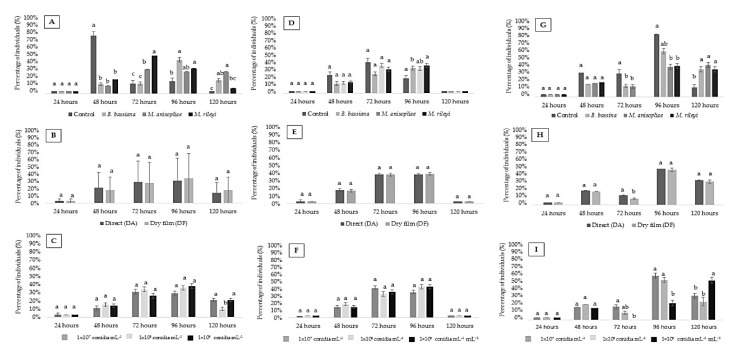
Percentage of *Chrysoperla externa* first, second and third instar (larval) period (% ± standard error) that changed their instar in each larval period after application of entomopathogenic fungi *Beauveria bassiana*, *Metarhizium anisopliae* and *Metarhizium rileyi* (T: 25 ± 1 °C, RH 70 ± 10% and 12 h photoperiod (light/dark)). (**A**–**C**) Individuals who changed from first to second urge, factor = P (products), A (application methods) and C (concentrations), respectively. (**D**–**F**) Individuals who changed from second to third instar, factor = P (products), A (application methods) and C (concentrations), respectively. (**G**–**I**) Individuals who changed from third instar to the pre-pupa, factor = P (products), A (application methods) and C (concentrations), respectively. Values (%) followed by equal letters at each assessment time do not differ from each according to a Tukey test at 5% probability.

Most larvae changed their instar between 48 and 120 HAA, influenced only by the products (P) (*p* < 0.05; Figure 2A). At 48 HAA, the control treatment provided the largest number of insects that changed stage, from the third instar to pupal stage (73%) compared with the other treatments. At 72 HAA, a higher percentage (48%) of larvae infected with the fungus *M. rileyi* showed a change of stage compared with the other treatments, followed by treatment with *M. anisopliae* (31%); both showed significant differences compared with the control and *B. bassiana* (13%). At 96 HAA, a smaller percentage (16%) of the larvae of the control treatment showed a change of stage than *B. bassiana* (43%) and *M. rileyi* (32%). At 120 HAA, a larger percentage of larvae treated with *B. bassiana* (17%) and *M. anisopliae* (28%) experienced a change of stage than the control (3%). However, *M. anisopliae* showed a higher percentage (28%) of instar change compared to the control (3%) and *M. rileyi* (7%) (Figure 2A).

Regarding the concentrations (C), only 1 × 10^8^ conidia mL^−1^ provided a reduction in the percentage of insects that presented a change of stage (*p*-value < 0.05; Figure 2C).

For the second instar, the change to the subsequent larval stage was more intense between 72 and 96 HAA. Treatment with *B. bassiana* provided a lower rate of change from the second to third instar than the control treatment. The *M. rileyi* treatment revealed a higher percentage of larvae that underwent a change in stage than the control treatment at 96 HAA (Figure 2D–F).

At each evaluation time, it was noticed that the larvae of the control treatment began the process of changing their larval state beginning at 72 HAA, with a higher average than the other fungal strains (P). In this period, variations were also observed for the factor application methods, and DA had a higher average than DF. The concentration of 1 × 10^9^ conidia mL^−1^ had a lower average than the concentration of 1 × 10^7^ conidia mL^−1^ (Figure 2G–I).

### 3.3. Duration (Days) Pupal Period

The third instar larvae of *C. externa* that survived the treatments began transitioning to pre-pupae as from 72 HAA. All larvae reached the pre-pupae stage at 120 HAA with the influence of the products (P), application methods (A) and concentrations (C) (*p*-value < 0.05; Supplementary Table S7).

All insects that survived and transformed in pupae made their cocoons between 148 and 164 HAA, with significant differences only for the products (P) (*p*-value < 0.05; Supplementary Table S6). There was no influence of products (P), application methods (A), concentrations (C) or interactions (P × A, P × C, A × C and P × A × C) on the pupal stage (*p*-value < 0.05; Supplementary Table S7).

**Figure 3:**
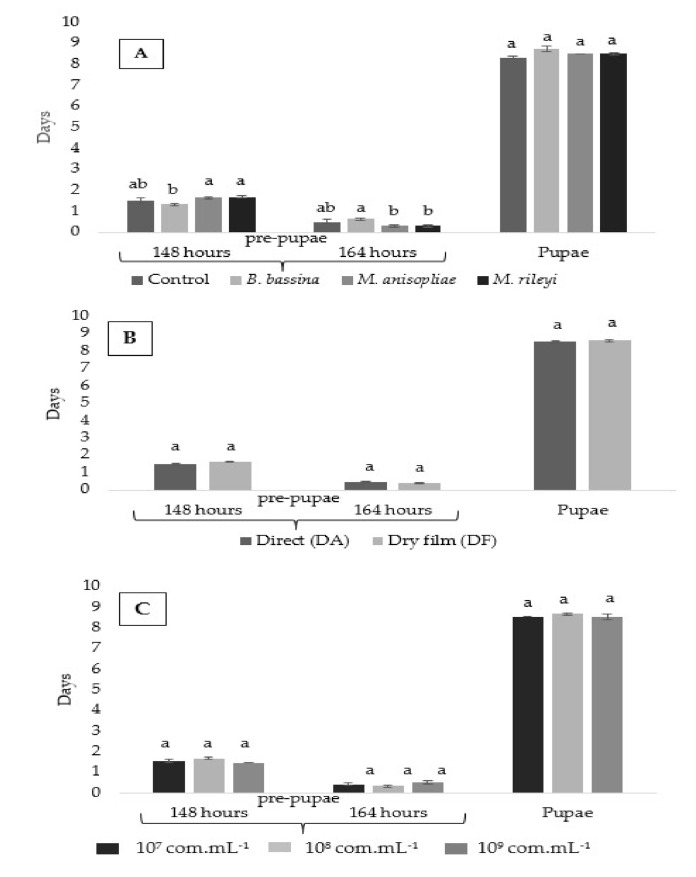
Average number (mean ± standard error) of the duration of pre-pupae and pupae of *Chrysoperla externa* after application of the entomopathogenic fungi *Beauveria bassiana*, *Metarhizium anisopliae* and *Metarhizium rileyi* (T: 25 ± 1 °C, RH of 70 ± 10% and photoperiod of 12 h (light/dark)). Duration in days for each stage according to factors: (**A**) product, (**B**) method of application and (**C**) concentrations. Means followed by equal letters at each assessment time do not differ from each according to a Tukey test at 5% probability.

At 96 HAA, the larvae treated with *M. anisopliae* and *M. rileyi* had a lower average duration of pre-pupae stage than those with the control treatment; the concentration of 1 × 10^9^ conidia mL^−1^ had a lower mean variation in the larval period than the other treatments (Figure 3A). At 120 HAA, all insects had concluded the pre-pupae stage, the control treatment had a lower mean than the entomopathogenic fungi and the concentration of 1 × 10^9^ conidia mL^−1^ had a higher mean than the other concentrations (Figure 3C).

From 148 to 164 HAA, all lacewings reached the pupal stage; in this period, a higher duration was recorded for larvae treated with *B. bassiana* than *M. anisopliae* and *M*. *rileyi*. The duration of the pupal period did not vary in relation to the treatments applied to the third-instar (larval) period of *C. externa*. (Figure 3A–C).

The pupae had 100% viability, confirmed by the emergence of all adult individuals. The adults showed no visual morphological deformities, and all had whole antennas, compound eyes, full abdomen and sizes between 21 and 23 mm (Supplementary Figure S5A–C).

## 4. Discussion

The low mortality rate of *C. externa* larvae treated with *M. anisopliae* (ESALQ E9) and *M. rileyi* (UFMS 03) indicate selectivity of these isolates in green lacewing larvae. These results corroborate the selectivity of *M. anisopliae* (E9) reported on the first instar (larval) period of *C. cincta* [31].

The mortality rate in the larval stage of *C. externa* due to the fungus *B. bassiana* (ESALQ PL63) was observed at 120 HAA. This period can be considered suitable for the colonization of the lacewing larvae, since the germination of conidia on the host usually occurs 12 h after inoculation, with the penetration of hemocele in 24 h, colonization occurring between three to five days and death occurring from six to seven days post-infection [42].

In Petri dishes that received treatment with *B. bassiana*, after drying, the formation of a white-colored adhesive film on the conidia was observed due to the excess moisture. This adhesive film maintained the artificial diet’s adherence to the surface of the Petri dishes and reduced the larvae mobility. The susceptibility of the larval stage of *C. externa* to the fungus *B. bassiana* is possibly related to the adhesive that the treatment provided on the Neuroptera exoskeleton. In this case, the product BOVERIL possibly favored the adherence and penetration of the conidia on the cuticle of the *C. externa* larvae. The mode of infection of the entomopathogenic fungi is via integument and involves processes of adherence and penetration of the pathogen on the insect cuticle, overcoming physical and chemical barriers to later cause the germination [43,44,45,46]. Previous studies have shown that the interaction of adjuvants with entomopathogenic fungi *Isaria fumosorosea* (strain ESALQ-1296) and *B. bassiana* (strain ESALQ-PL63) enhances the pathogen action on the Asian citrus psyllid *Diaphorina citri* Kuwayama (Hemiptera, Liviidae) upon the physical and mechanical processes of infection by the integument [47,48]. The selectivity of the entomopathogenic fungi to entomophagous insects depends on several factors, among them, the formulation of emulsifiers, adjuvants and oils [46].

Although there was higher mortality for *B. bassiana*-infected larvae, at 120 HAA the percentages obtained in the present study were lower than 30%. Studies with entomopathogens consider the pathogen when it reaches 40% host death [49,50,51,52]. By considering the pathogenic action of these fungi on green lacewing larvae, we can assume selectivity regarding the survival of individuals in the second and third instar.

The findings of the present study corroborate results obtained by other studies that showed selectivity or compatibility of *B. bassiana* isolates with low mortality rates (<30%) on green lacewing species such as the first instar (larval) period of *Chrysopa exterior* Navás (Neuroptera, Chrysopidae) [53], the third instar (larval) period of *C. externa* [30] and the larval stage of *C. externa* [54].

Although the present study evaluated three different concentrations (1 × 10^7^, 1 × 10^8^ and 1 × 10^9^ conidia mL^−1^) for each of the entomopathogenic fungi, the mortality of *C. externa* was not influenced by an increase of the concentrations. Studies with the third instar (larval) period of *Chrysoperla kolthoffi* Navás (1927) (Neuroptera: Chrysopidae) treated with *M. anisopliae* var. *anisopliae* showed an increase of mortality starting from the concentration of 1.5 × 10^8^ conidia mL^−1^ and 100% mortality for concentrations higher than 1.5 × 10^12^ conidia mL^−1^ [55]. In general, suspensions of entomopathogenic fungi did not affect the development of the biological cycle of *C. externa*; for those that presented the pupal phase and later, 100% of adults emerged. The studies with *M. anisopliae* var. *anisopliae* in early stage *C. kolthoffi* larvae showed that 75.8% of emerging adults were treated with the lowest concentrations (1.5 × 10^4^ and 1.5 × 10^7^ conidia mL^−1^) of the fungus [56].

Variations in the duration of larval, pre-pupae and pupae stages were not related to treatments with entomopathogenic fungi, concentrations or application methods, as there were also variations of these stages in the control insects. The variations in the duration of the larval stages are possibly related to the metabolism of each larva and the food consumption during each stage. The changes of instars of chrysopids are associated with the quantity and quality of prey consumed to meet the nutrient needs of each larval stage because all the reserves of the larval stage will be used in the prepupae and pupae stage and in the initial reproduction phase [11]. Generally, the lowest larval period was recorded for the second instar, a fact that can be attributed to the biological characteristics of the species [57,58].

The results obtained from this study evidence that the biological development of larvae of *C. externa* was not influenced by the fungus *B. bassiana* (strain ESALQ PL63), *M. anisopliae* (strain ESALQ E9) or *M. rileyi* (strain UFMS 03). These species of fungi can be recommended, aiming at the management of populations of arthropod pests, with a low effect on *C. externa* when it is present in the agrosystem.

These results indicate that there are possibilities for new research to validate the selectivity of these strains concerning the other development stages of *C. externa*. Thus, it is necessary to carry out studies to verify the effect of other strains or biobased products on the development of different species of green lacewings for various regions of Brazil.

New investigations are justified due to the high diversity of fungal strains from Brazil and the importance of determining factors for their mortality, such as the genetic variability of each isolate [59,60], the behavior and physiological condition of the host [61], the defense mechanisms used by insects [44,62,63,64,65], the lethal dose [66] and the compatibility and interaction with the entomophagous insects [30,54,66,67].

## 5. Conclusions

There was no effect of fungi concentrations or application methods on all larval stages of *C. externa*. Although the *B. bassiana* strain induced higher mortality than *M. anisopliae* and *M. rileyi* strains on larvae of *C. externa*, these three entomopathogenic fungi may be used in association with *C. externa* for sustainable pest management.

## Supplementary Materials

The following are available online at https://www.mdpi.com/2075-4450/11/10/716/s1, Figure S1: Mortality of entomopathogens biossay on the larval stage of *Chrysoperla externa*. (A) first instar of *Chrysoperla externa*; (B) second instar of *Chrysoperla externa*; (C) third instar of *Chrysoperla externa*. (Images recorded with 13 Mpx camera. Surfboard (A, B and C), with the aid of a stereoscopic microscope with a magnification of 20×). Figure S2: Mortality Confirmed. (A) Larva of *Chrysoperla externa* dead after 120 h, treatment with *B. bassiana*; (B) larvae after the surface disinfection process; (C) Larvae over humid chamber containing sterile cotton moistened with distilled water to provide mycelial growth; (D) Storage of humid chambers in B.O.D. at 25 ± 1 °C, (RH) 70 ± 10% and 12 h photoperiod (L: D). (Images recorded with 13 Mpx camera. With the aid of a stereoscopic microscope with a magnification of 20×). Figure S3: Visual scale for viability or deformity of *Chrysoperla externa*. (A and B) Insects with wing and antenna deformity patterns; (C) Viable insect. (Images recorded with 13 Mpx camera. With the aid of a stereoscopic microscope with a magnification of 20×). Figure S4: Conidiogenesis of *Beauveria bassiana* (ESALQ PL63 strain), on cadavers of *Chrysoperla externa* with mortality at 120 hours after application. (A) confirmed mortality by the fungus *Beauveria bassiana* (1 × 10^7^ con·mL^−1^), (D.A.), on dead bodies of second instar of *Chrysoperla externa*, 5 days after mortality; (B) Conidiogenesis on third instar larvae, 3 days after the death (1 × 10^8^ con·mL^−1^), (D.O.); (C) conidiogenesis of *Beauveria bassiana* (1 × 10^9^ con·mL^−1^), (D.A.), on dead bodies of third instar, 3 days 39 after death. (Images recorded with 13 Mpx camera. With the aid of a stereoscopic microscope with a 40 magnification of 20×). Figure S5: Emergence of *Chrysoperla externa* after application of treatments on third instar larvae. (A) The emergence of C. externa, treatment with *B. bassiana* (1 × 10^9^ conidia mL^−1^) (D.F.); (B) Imago of *C. externa*, treatment with *M. anisopliae* (1 × 10^9^ conidia·mL^−1^) (D.F.), highlighted the antennas and the golden eyes, characteristic of the group green lacewing; (C) adults of *C. externa*, regular size, control treatment (D.A.). (Images recorded with 13 Mpx camera. With the aid of a stereoscopic microscope with a magnification of 20×). Table S1: Summary of ANOVA (values of mean square) of the mortality of first instar (larval) period of *C. externa* after application of entomopathogenic fungi *B. bassiana*, *M. anisopliae* and *M. rileyi* (T: 25 ± 1 °C, RH of 70 ± 10% and photoperiod of 12 h (L:D)). Table S2: Summary of ANOVA (values of mean square) of the mortality of second instar (larval) period of *C. externa* after application of entomopathogenic fungi *B. bassiana*, *M. anisopliae* and *M. rileyi* (T: 25 ± 1 °C, RH of 70 ± 10% and photoperiod of 12 h (L:D)). Table S3: Summary of ANOVA (values of mean square) of the mortality of third instar (larval) period of *C. externa* after application of entomopathogenic fungi *B. bassiana*, *M. anisopliae* and *M. rileyi* (T: 25 ± 1 °C, RH of 70 ± 10% and photoperiod of 12 h (L:D)). Table S4: Summary of ANOVA (values of mean square) of the duration of first instar (larval) period of *C. externa* after application of the entomopathogenic fungi *B. bassiana*, *M. anisopliae* and *M. rileyi* (T: 25 ± 1 °C, RH of 70 ± 10% and photoperiod of 12 h (L:D)). Table S5: Summary of ANOVA (values of mean square) of the duration of second instar (larval) period of *C. externa* after application of the entomopathogenic fungi *B. bassiana*, *M. anisopliae*, and *M. rileyi* (T: 25 ± 1 °C, RH of 70 ± 10% and photoperiod of 12 h (L:D)). Table S6: Summary of ANOVA (values of mean square) of the duration of third instar (larval) period of *C. externa* after application of the entomopathogenic fungi *B. bassiana*, *M. anisopliae*, *M. rileyi* (T: 25 ± 1 °C, RH of 70 ± 10% and photoperiod of 12 h (L:D)). Table S7: Summary of ANOVA (values of mean square) of the duration of third instar (larval) period and pupae of *C. externa* after application of the entomopathogenic fungi *B. bassiana*, *M. anisopliae*, *M. rileyi* (T: 25 ± 1 °C, RH of 70 ± 10% and photoperiod of 12 h (L:D)).
