# Supplementary material for: Selectivity of Entomopathogenic Fungi to Chrysoperla externa (Neuroptera: Chrysopidae)
Source: Insects. 2020 Oct 19;11(10):716. doi: 10.3390/insects11100716 (PMC7603232; doi:10.3390/insects11100716)
Supplement: Supplementary file 1 [file insects-11-00716-s001.pdf]

insects

MDPI

Article

Selectivity of Entomopathogenic Fungi to *Chrysoperla externa* (Neuroptera: Chrysopidae)

Pamella Mingotti Dias [1], Elisângela de Souza Loureiro [1,2,3,*], Luis Gustavo Amorim Pessoa [2,3], Gabriel Luiz Reis Devoz [2], Gilson Bárbaro Barbosa Junior [3], Allan Macali Werner [3], Acacio Aparecido Navarrete [3] and Paulo Eduardo Teodoro [2,3]

1 Graduate Program in Entomology and Biodiversity Conservation, Federal University of Grande Dourados, Dourados 79.804-970, Brazil; pamellamingotti@hotmail.com

2 Agronomy, Federal University of Mato Grosso do Sul, Chapadão do Sul 79.560-000, Brazil; luis.pessoa@ufms.br (L.G.A.P.); gabriel.devoz@gmail.com (G.L.R.D.); paulo.teodoro@ufms.br (P.E.T.)

3 Graduate Program in Agronomy, Area of Concentration in Crop Science, Federal University of Mato Grosso do Sul, Chapadão do Sul 79.560-000, Brazil; gilson.barbarobarbosa@gmail.com (G.B.B.J.); allanwerner@hotmail.com (A.M.W.); acacionavarrete@gmail.com (A.A.N.)

* Correspondence: elisangela.loureiro@ufms.br

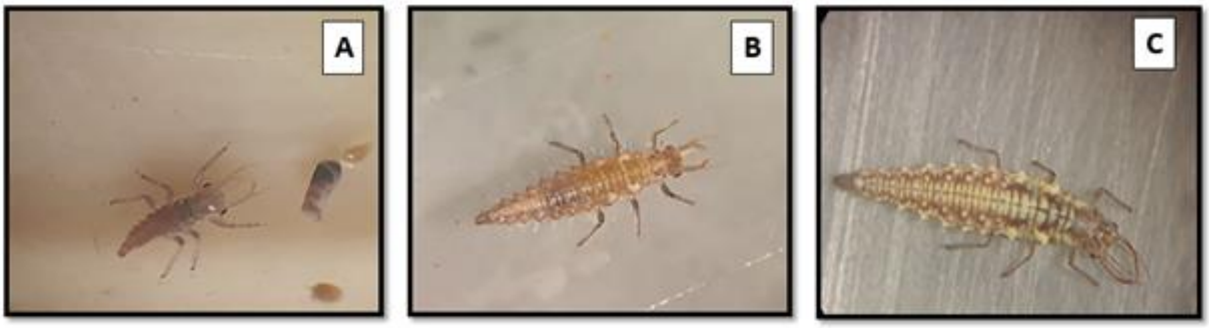

Figure S1: Mortality of entomopathogens biossay on the larval stage of *Chrysoperla externa*. (**A**) first instar of *Chrysoperla externa*; (**B**) second instar of *Chrysoperla externa*; (**C**) third instar of *Chrysoperla externa.* (Images recorded with 13 Mpx camera. Surfboard (**A**, **B** and **C**), with the aid of a stereoscopic microscope with a magnification of 20×).

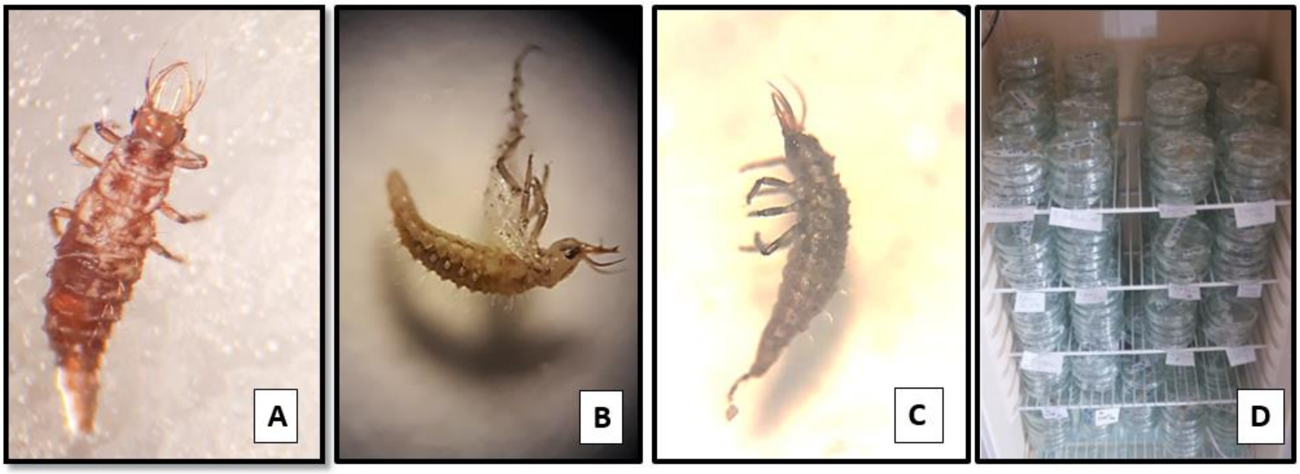

Figure S2: Mortality Confirmed. (**A**) Larva of *Chrysoperla externa* dead after 120 h, treatment with *B. bassiana*; (**B**) larvae after the surface disinfection process; (**C**) Larvae over humid chamber containing sterile cotton moistened with distilled water to provide mycelial growth; (**D**) Storage of humid chambers in B.O.D. at 25 ± 1 °C, (RH) 70 ± 10% and 12 hour photoperiod (L:D). (Images recorded with 13 Mpx camera. With the aid of a stereoscopic microscope with a magnification of 20×).

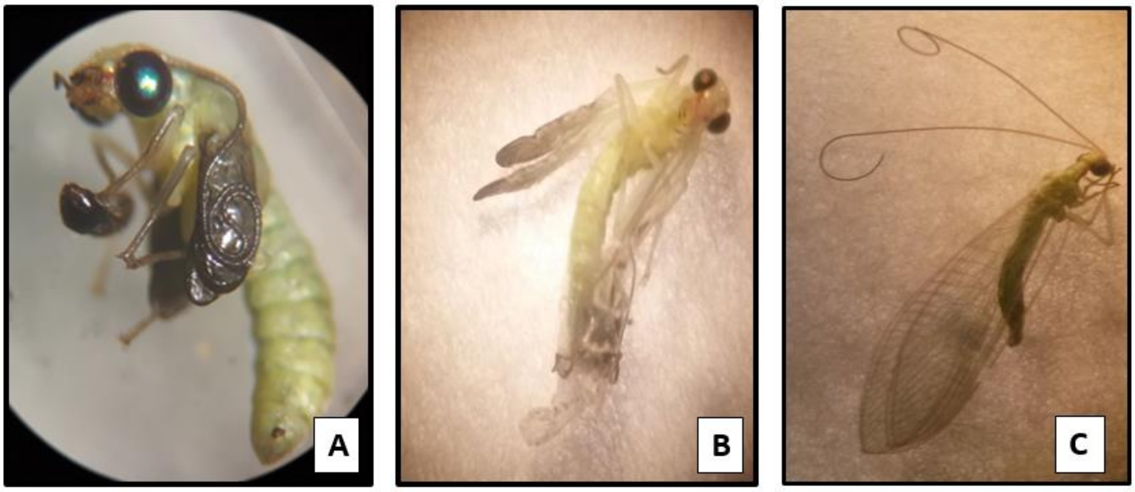

Figure S3: Visual scale for viability or deformity of *Chrysoperla externa*. (**A** and **B**) Insects with wing and antenna deformity patterns; (**C**) Viable insect. (Images recorded with 13 Mpx camera. With the aid of a stereoscopic microscope with a magnification of 20×).

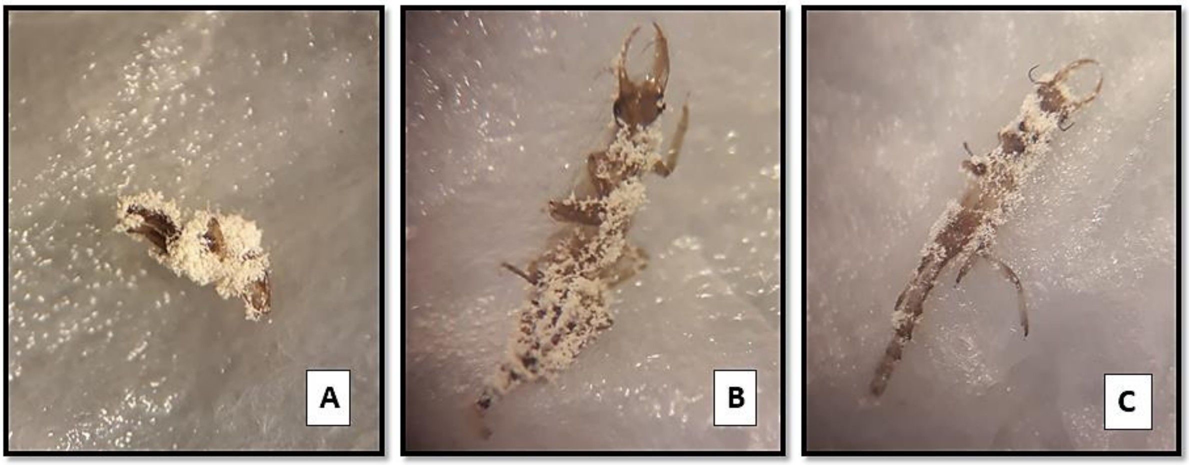

Figure S4: Conidiogenesis of *Beauveria bassiana* (ESALQ PL63 strain), on cadavers of *Chrysoperla externa* with mortality at 120 hours after application. (**A**) confirmed mortality by the fungus *Beauveria bassiana* ($1x10^7$ con.mL^{-1}), (D.A.), on dead bodies of second instar of *Chrysoperla externa*, 5 days after mortality; (**B**) Conidiogenesis on third instar larvae, 3 days after the death ($1x10^8$ con.mL^{-1}), (D.O.); (**C**) conidiogenesis of *Beauveria bassiana* ($1x10^9$ con.mL^{-1}), (D.A.), on dead bodies of third instar, 3 days after death. (Images recorded with 13 Mpx camera. With the aid of a stereoscopic microscope with a magnification of 20×).

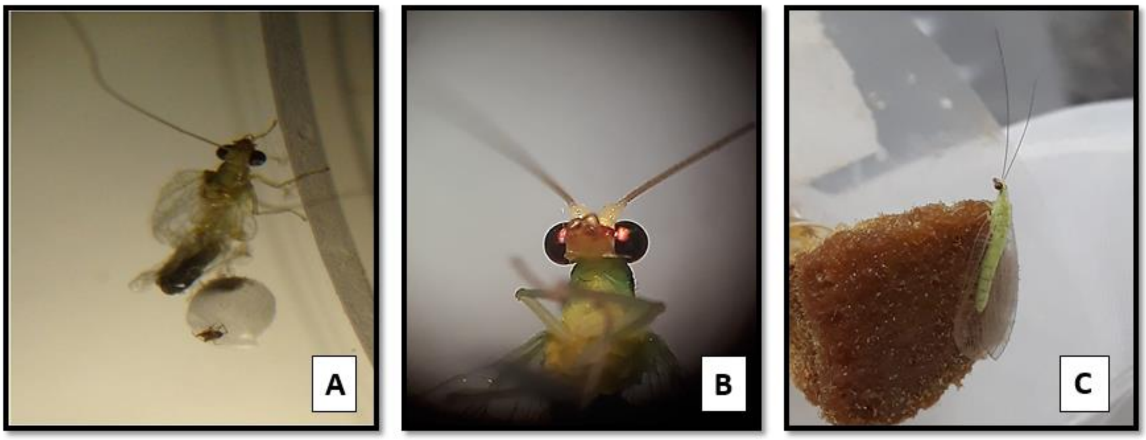

Figure S5: Emergence of *Chrysoperla externa* after application of treatments on third instar larvae. (**A**) The emergence of *C. externa*, treatment with *B. bassiana* ($1x10^9$ conidia mL^{-1}) (D.F.); (**B**) Imago of *C. externa*, treatment with *M. anisopliae* ($1x10^9$ conidia.mL^{-1}) (D.F.), highlighted the antennas and the golden eyes, characteristic of the group green lacewing; (**C**) adults of *C. externa*, regular size, control treatment (D.A.). (Images recorded with 13 Mpx camera. With the aid of a stereoscopic microscope with a magnification of 20×).

3.1. *Mortality of C. externa* Larvae

Table S1: Summary of ANOVA (values of mean square) of the mortality of first instar larvae of *C. externa* after application of entomopathogenic fungi *B. bassiana*, *M. anisopliae* and *M. rileyi* (T: 25 ± 1 °C, RH of 70 ± 10% and photoperiod of 12 hours (L:D)).

SV	DF	24 hours	48 hours	72 hours	96 hours	120 hours
Product (P)	3	0.00 ns	0.00 ns	0.00 ns	0.00 ns	1.40*
Application (A)	1	0.00 ns	0.00 ns	0.00 ns	0.00 ns	0.08 ns
Concentration (C)	3	0.00 ns	0.00 ns	0.00 ns	0.00 ns	0.21 ns
PxA	3	0.00 ns	0.00 ns	0.00 ns	0.00 ns	0.03 ns
PxC	9	0.00 ns	0.00 ns	0.00 ns	0.00 ns	0.02 ns
AxC	3	0.00 ns	0.00 ns	0.00 ns	0.00 ns	0.00 ns
PxAxC	9	0.00 ns	0.00 ns	0.00 ns	0.00 ns	0.01 ns
Residue	580	0.03	0.03	0.03	0.03	0.01

* significant at 5% probability by F-test; ns: not-significant at 5% probability by F-test. SV: source of variation; DF: degrees of freedom; Numbers are F-values.

Table S2: Summary of ANOVA (values of mean square) of the mortality of second instar larvae of *C. externa* after application of entomopathogenic fungi *B. bassiana*, *M. anisopliae* and *M. rileyi* (T: 25 ± 1 °C, RH of 70 ± 10% and photoperiod of 12 hours (L:D)).

SV	DF	24 hours	48 hours	72 hours	96 hours	120 hours
Product (P)	3	0.00 ns	0.00 ns	0.00 ns	0.00 ns	0.80*
Application (A)	1	0.00 ns	0.00 ns	0.00 ns	0.00 ns	0.01 ns
Concentration (C)	3	0.00 ns	0.00 ns	0.00 ns	0.00 ns	0.01 ns
PxA	3	0.00 ns	0.00 ns	0.00 ns	0.00 ns	0.00 ns
PxC	9	0.00 ns	0.00 ns	0.00 ns	0.00 ns	0.01 ns
AxC	3	0.00 ns	0.00 ns	0.00 ns	0.00 ns	0.01 ns
PxAxC	9	0.00 ns	0.00 ns	0.00 ns	0.00 ns	0.06 ns
Residue	580	0.03	0.03	0.03	0.03	0.07

* significant at 5% probability by F-test; ns: not-significant at 5% probability by F-test. SV: source of variation; DF: degrees of freedom; Numbers are F-values.

Table S3: Summary of ANOVA (values of mean square) of the mortality of third instar larvae of *C. externa* after application of entomopathogenic fungi *B. bassiana*, *M. anisopliae* and *M. rileyi* (T: 25 ± 1 °C, RH of 70 ± 10% and photoperiod of 12 hours (L:D)).

SV		24 hours	48 hours	72 hours	96 hours	120 hours
**Product (P)**	3	0.00 ns	0.00 ns	0.00 ns	0.00 ns	0.15*
**Application (A)**	1	0.00 ns	0.00 ns	0.00 ns	0.00 ns	0.41 ns
**Concentration (C)**	3	0.00 ns	0.00 ns	0.00 ns	0.00 ns	0.46 ns
**PxA**	3	0.00 ns	0.00 ns	0.00 ns	0.00 ns	0.01 ns
**PxC**	9	0.00 ns	0.00 ns	0.00 ns	0.00 ns	0.03 ns
**AxC**	3	0.00 ns	0.00 ns	0.00 ns	0.00 ns	0.00 ns
**PxAxC**	9	0.00 ns	0.00 ns	0.00 ns	0.00 ns	0.18 ns
**Residue**	580	0.03	0.03	0.03	0.03	0.05

* significant at 5% probability by F-test; ns not-significant at 5% probability by F-test. SV: source of variation; DF: degrees of freedom; Numbers are F-values.

3.2. Change of Stage of C. externa Larvae

Table S4: Summary of ANOVA (values of mean square) of the duration of first instar larvae of *C. externa* after application of the entomopathogenic fungi *B. bassiana, M. anisopliae* and *M. rileyi* (T: 25 ± 1 °C, RH of 70 ± 10% and photoperiod of 12 hours (L:D)).

SV	DF	24 hours	48 hours	72 hours	96 hours	120 hours
Product (P)	3	0.00 ns	6.59*	4.35*	1.27*	1.65*
Application (A)	1	0.00 ns	0.13 ns	0.01 ns	0.10 ns	0.20 ns
Concentration (C)	3	0.00 ns	0.05 ns	0.23 ns	0.27 ns	0.48*
PxA	3	0.00 ns	0.12 ns	0.34 ns	0.18 ns	0.24 ns
PxC	9	0.00 ns	0.12 ns	0.46 ns	0.28 ns	0.11 ns
AxC	3	0.00 ns	0.02 ns	0.11 ns	0.18 ns	0.15 ns
PxAxC	9	0.00 ns	0.03 ns	0.04 ns	0.05 ns	0.06 ns
Residue	580	0.03	0.12	0.17	0.21	0.12

* significant at 5% probability by F-test; ns: not-significant at 5% probability by F-test. SV: source of variation; DF: degrees of freedom; Numbers are F-values.

Table S5: Summary of ANOVA (values of mean square) of the duration of second instar larvae of *C. externa* after application of the entomopathogenic fungi *B. bassiana, M. anisopliae,* and *M. rileyi* (T: 25 ± 1 °C, RH of 70 ± 10% and photoperiod of 12 hours (L:D)).

SV	GL	24 hours	48 hours	72 hours	96 hours	120 hours
Product (P)	3	0.00 ns	0.26 ns	0.71*	0.63*	0.00 ns
Application (A)	1	0.00 ns	0.02 ns	0.00 ns	0.02 ns	0.00 ns
Concentration (C)	3	0.00 ns	0.06 ns	0,21 ns	0.24 ns	0.00 ns
PxA	3	0.00 ns	0.25 ns	0.39 ns	0.24 ns	0.00 ns
PxC	9	0.00 ns	0.10 ns	0.09 ns	0.04 ns	0.00 ns
AxC	3	0.00 ns	0.11 ns	0.01 ns	0.15 ns	0.00 ns
PxAxC	9	0.00 ns	0.07 ns	0.10 ns	0.07 ns	0.00 ns
Residue	580	0.03	0.14	0.23	0.23	0.03

* significant at 5% probability by F-test; ns: not-significant at 5% probability by F-test. SV: source of variation; DF: degrees of freedom; Numbers are F-values.

Table S6: Summary of ANOVA (values of mean square) of the duration of third instar larvae of *C. externa* after application of the entomopathogenic fungi *B. bassiana, M. anisopliae, M. rileyi* (T: 25 ± 1 °C, RH of 70 ± 10% and photoperiod of 12 hours (L:D)).

SV	DF	24 hours	48 hours	72 hours	96 hours	120 hours
Product (P)	3	0.00 ns	0.00 ns	1.21*	2.97*	4.12*
Application (A)	1	0.00 ns	0.00 ns	0.32*	0.02 ns	1.50 ns
Concentration (C)	3	0.00 ns	0.00 ns	0.72*	3.68*	1.54*
PxA	3	0.00 ns	0.00 ns	0.20 ns	0.00 ns	1.07 ns
PxC	9	0.00 ns	0.00 ns	0.12 ns	1.07 ns	0.47 ns
AxC	3	0.00 ns	0.00 ns	0.06 ns	0.12 ns	1.43 ns
PxAxC	9	0.00 ns	0.00 ns	0.03 ns	0.19 ns	0.42 ns
Residue	580	0.03	0.03	0.07	0.20	0.64

* significant at 5% probability by F-test; ns: not-significant at 5% probability by F-test. SV: source of variation; DF: degrees of freedom; Numbers are F-values.

Table S7: Summary of ANOVA (values of mean square) of the duration of third instar larvae and pupae of *C. externa* after application of the entomopathogenic fungi *B. bassiana*, *M. anisopliae*, *M. rileyi* (T: 25 ± 1 °C, RH of 70 ± 10% and photoperiod of 12 hours (L:D)).

**SV**	**DF**	**148 hours**	**164 hours**	**Pupae**
Product (P)	3	4.98*	4.12*	3.44 ^{ns}
Application (A)	1	2.40 ^{ns}	1.50 ^{ns}	0,54 ^{ns}
Concentration (C)	3	1.26 ^{ns}	0.87 ^{ns}	0.75 ^{ns}
PxA	3	1.36 ^{ns}	1.07 ^{ns}	0.28 ^{ns}
PxC	9	0.57 ^{ns}	0.47 ^{ns}	0.52 ^{ns}
AxC	3	1.34 ^{ns}	1.43 ^{ns}	1.27 ^{ns}
PxAxC	9	0.28 ^{ns}	0.42 ^{ns}	0.87 ^{ns}
Residue	580	0.65	0.64	1.68

* significant at 5% probability by F-test; ns: not-significant at 5% probability by F-test. SV: source of variation; DF: degrees of freedom; Numbers are F-values.

© 2020 by the authors. Submitted for possible open access publication under the terms and conditions of the Creative Commons Attribution (CC BY) license (http://creativecommons.org/licenses/by/4.0/).
